# Supplementary material for: The Critical Role of 12-Methyl Group of Anthracycline Dutomycin to Its Antiproliferative Activity
Source: Molecules. 2022 May 23;27(10):3348. doi: 10.3390/molecules27103348 (PMC9144609; doi:10.3390/molecules27103348)
Supplement: Supplementary file 1 [file molecules-27-03348-s001.zip › molecules-1682839-SI.pdf]

Supplementary Material

S1 1H NMR spectrum of dutomycin

The compound obtained through liquid phase purification was removed the water vapor with a vacuum drying oven one night in advance, and the obtained dry powder was dissolved with a deuterated chloroform solvent. The hydrogen spectrum was obtained by scanning with a nuclear magnetic resonance apparatus, and the obtained substance was confirmed to be our target compound dutomycin by comparison.

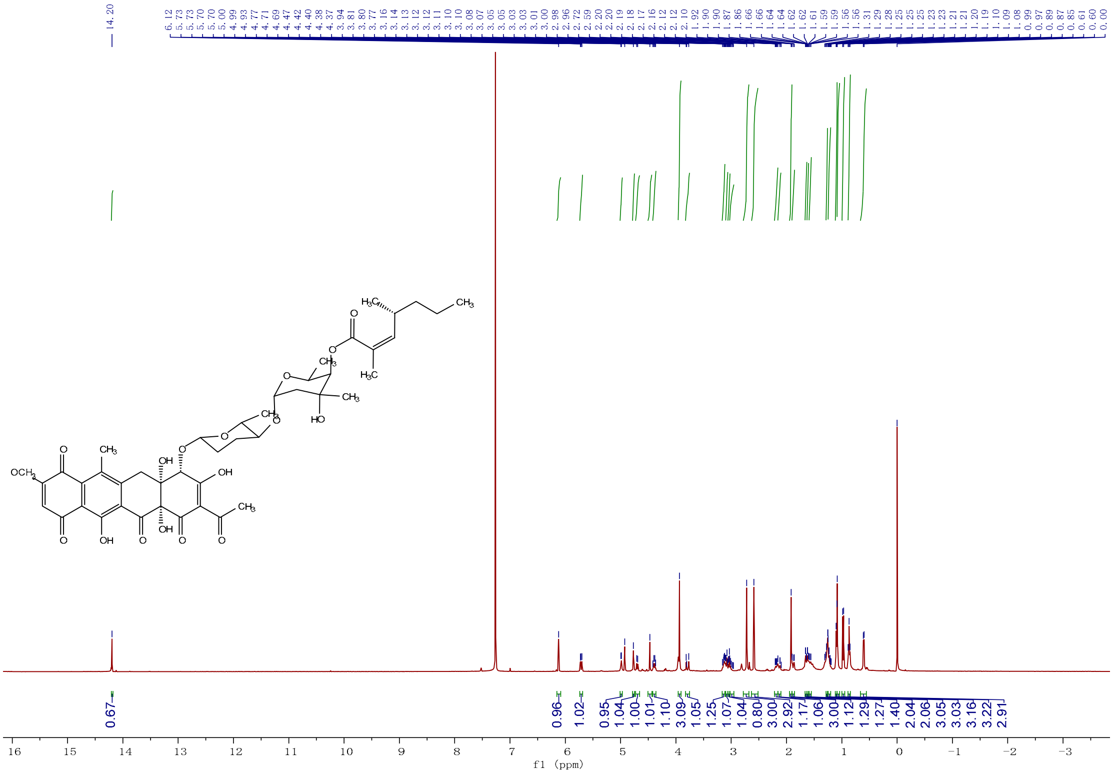

Figure S1: 1H NMR spectrum of dutomycin

S2 1H NMR spectrum of SW91

The compound obtained through liquid phase purification was removed one night in advance with a vacuum drying oven to remove water vapor, the obtained dry powder was dissolved in deuterated chloroform solvent, and the hydrogen spectrum was obtained by scanning with NMR.

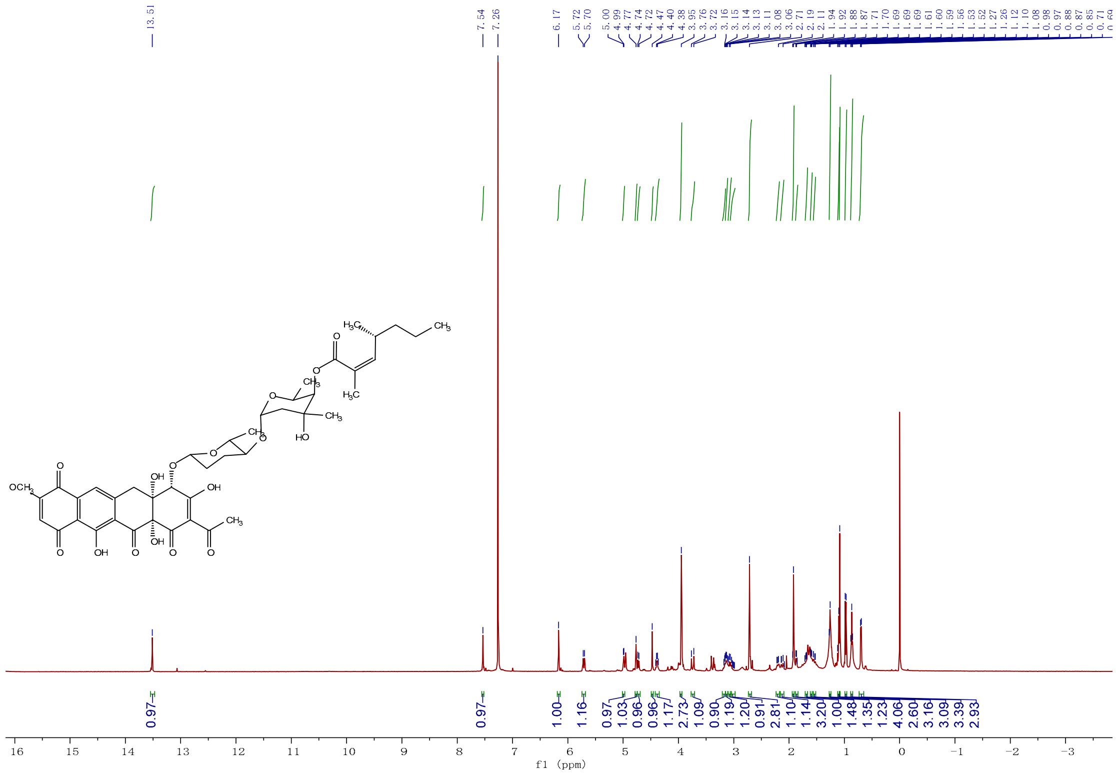

Figure S2: 1H NMR spectrum of SW91

Supplementary Figures

S3 Histogram of viability of doxorubicin in different cell lines

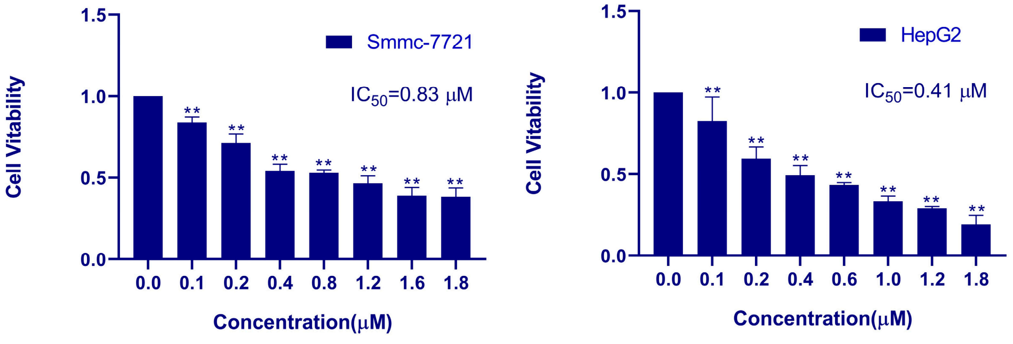

(**a**) (**b**)

Figure S3: Histogram of viability of doxorubicin in different cell lines. (a) Histogram of viability in Smmc-7721 cells. (b) Histogram of viability in HepG2 cells.

S4 Apoptosis map gate.

The apoptotic cell experiment is gated to obtain a cell population that we need to analyze, and the apoptosis situation is analyzed based on this cell population.

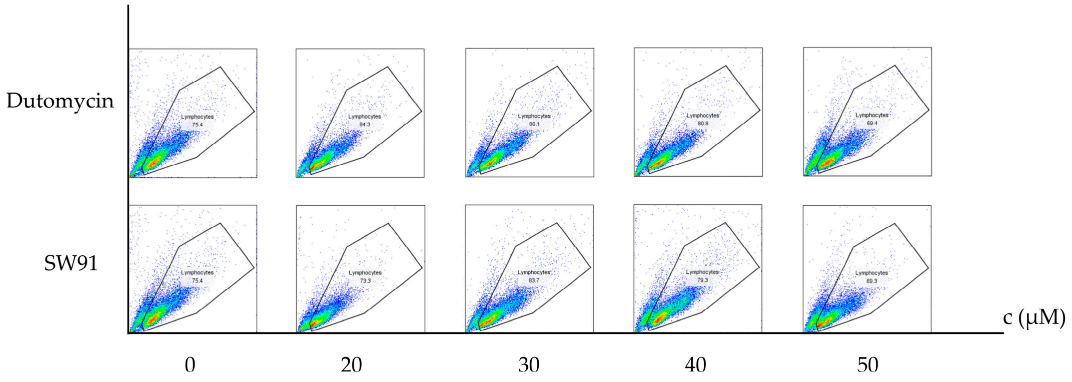

Figure S4: Apoptosis map gate.

S5 Apoptosis plot with indifferent concentration

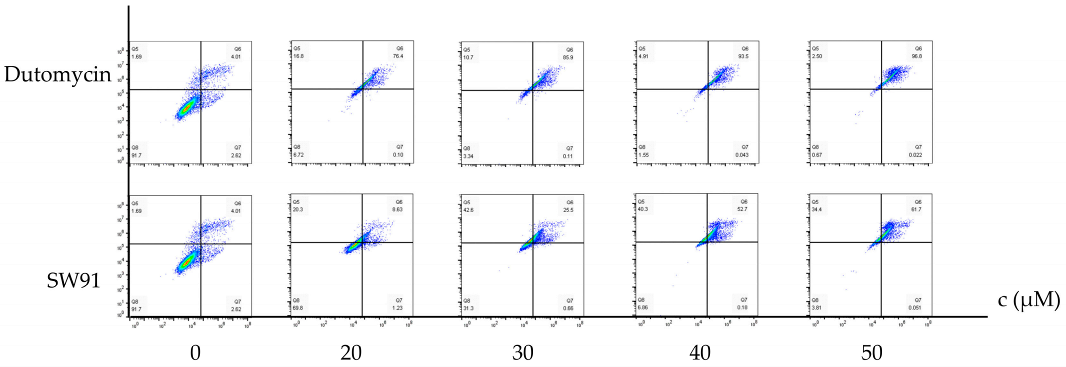

Figure S5: Apoptosis plot with indifferent concentration（μM）

S6 cell cycle gate

Using the cells obtained by the flow cytometer to gate, first obtain a cell group that we need to analyze, circle single cells based on this cell group and obtain our experimental data by analyzing the cycle of this group of single cells.

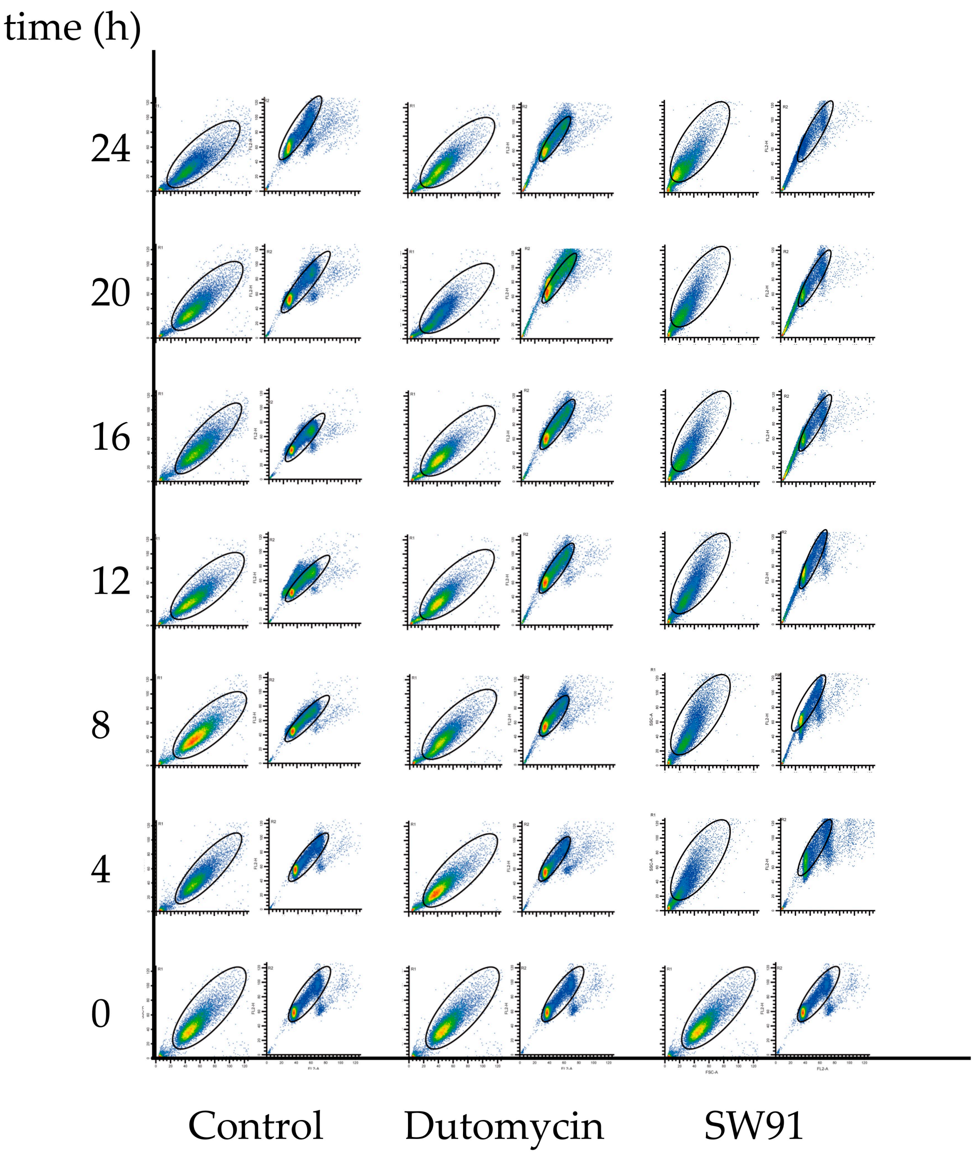

Figure S6: cell cycle gate.

Supplementary Tables

Table S1 ^{1}H-NMR (400MHz) data for dutomycin and SW91(Chloroform-d)

Position	^{1}H-NMR, δ, ppm (area, m, J in Hz)	
	dutomycin	SW91
3	6.12 (s, 1H)	6.17 (s, 1H)
5-OH	14.20 (s, 1H)	13.51 (s, 1H)
10	4.47 (s, 1H)	4.47(s, 1H)
11	3.79 (d, J = 17.7 Hz, 1H)	3.74 (d, J = 17.5 Hz, 1H)
	2.98 (m, 1H)	3.08 (m, 1H)
12		7.54 (s, 1H)

13	2.59 (s, 3H)	
15	2.72 (s, 3H)	2.71 (s, 3H)
16	3.94 (s, 3H)	3.95 (s, 3H)
1′	4.70 (d, J = 7.8 Hz, 1H)	4.73 (d, J = 8.0 Hz, 1H)
2′	1.56 (m, 1H)	1.54 (m, 1H)
	2.19 (m, J = 3.4 Hz, 1H)	2.21 (m, 1H)
3′	2.12 (m, 1H)	2.13 (m, 1H)
	1.62 (m, 1H)	1.69 (m, 1H)
4′	3.14 (m, 1H)	3.13 (m, 1H)
5′	3.05 (m, 1H)	3.02 (m, 1H)
6′	0.60 (d, J = 6.0 Hz, 3H)	0.70 (d, J = 6.0 Hz, 3H)
1′′	4.99 (d, 1H)	4.99 (d, J = 3.3 Hz, 1H),
2′′	1.88 (m, 1H)	1.87 (m, 1H)
	1.65 (m, 1H)	1.60(m, 1H)
4′′	4.77 (s, 1H)	4.77 (s, 1H)
5′′	4.39 (q, *J* = 6.4 Hz 1H)	4.39 (q, J = 6.1 Hz, 1H)
6′′	1.10 (d, J = 6.5 Hz, 3H)	1.11(d, 3H)
7′′	1.08 (s, 3H)	1.08 (d, J = 7.0 Hz, 3H)
3′′′	1.92 (s, 3H)	1.93 (d, 3H)
4′′′	5.71 (dd, 1H)	5.71 (d, J = 10.1 Hz, 1H)
5′′′	3.09 (m, 1H)	3.17 (m, 1H)
6′′′	0.98 (d, J = 6.6 Hz, 3H)	0.98 (d, J = 6.6 Hz, 3H)
7′′′	1.22 (m, 2H)	1.26 (m, J = 2.3 Hz, 2H)
8′′′	1.29 (m, 2H)	1.26 (m, J = 3.6 Hz, 2H)
9′′′	0.87 (t, 3H)	0.87 (t, J = 6.4 Hz, 3H)
